# Perception-Based Methods and Beyond: A Current Opinion on How to Assess Static Stretching Intensity

**DOI:** 10.1007/s40279-025-02307-1

**Published:** 2025-09-11

**Authors:** Konstantin Warneke, Anthony J. Blazevich, Daniel Jochum, David G. Behm, Ewan Thomas, Masatoshi Nakamura, José Afonso

**Affiliations:** 1 Institute of Human Movement Science and Exercise Physiology, Friedrich Schiller University Jena, Jena, Germany; 2 Institute of Psychology, Leuphana University Lüneburg, Lüneburg, Germany; 3 Department for Human Movement Science and Exercise Physiology, Institute for Sport Science, Seidelstraße 20, Jena, Germany; 4 Centre for Human Performance, School of Medical and Health Sciences, Edith Cowan University, Joondalup, WA Australia; 5 Department of Health Sciences and Technology, ETH Zurich, Zurich, Switzerland; 6 School of Human Kinetics and Recreation, Memorial University of Newfoundland, St. John’s, Canada; 7 Sport and Exercise Sciences Research Unit, Department of Psychology, Educational Science and Human Movement, University of Palermo, Palermo, Italy; 8 Faculty of Rehabilitation Sciences, Nishi Kyushu University, Kanzaki, Saga Japan; 9 Centre of Research, Education, Innovation, and Interventionin Sport (CIFI2D), Faculty of Sport, University of Porto, Porto, Portugal

## Abstract

Muscle stretching is widely used in clinical, athletic, and otherwise healthy populations, yet a consensual definition of stretch intensity—a key component of stretch load—does not exist. This is important because the effects of stretch intensity on range of motion and strength are controversial but suggested to affect clinical practice and scientific research. Most commonly, stretch intensity is defined in relation to an individual’s perceived level of discomfort or pain; however, these definitions are problematic for several reasons, including that consensual and objective quantifiable definitions of ‘pain’ and ‘discomfort’ do not exist, perceptions vary widely (and may not be sensed in some populations), and their ordinal (interval) nature is problematic from a statistical (research) point of view. The maximal range of motion or stretch distance may instead be useful; however, it can be difficult to define the ‘start of stretch’ and tissue stress varies non-linearly with range of motion or distance, meaning tissue load (stress) varies markedly with small changes in joint angle or distance near the stretch limit but varies less when stretches are performed further from it. Alternatively, setting joint angles or stretch distances as a percentage of the peak passive torque or resistive force can circumvent these issues, removing the need to define the ‘start of stretch’ and ensuring that intensity changes largely reflect changes in tissue load; however, torque/force measurement can sometimes be difficult or impossible to assess. A concerted research effort is thus required to produce an accepted definition of stretch intensity, and then to clarify how this can be quantified in scientific and practical settings.

## Key Points

**Table:** 

Stretching is often implemented as part of warm-up routines but also used longer term to improve muscle function and structure. However, quantification of stretching intensity in the literature is highly heterogeneous and far from consensual.
Stretch intensity is often quantified via pain perception but this approach must be questioned as results are not interval scaled and depend on subjective experiences of individuals.
A more objective approach, including passive peak torque and/or muscle stiffness evaluations, may be necessary to allow scientifically sound conclusions to be drawn about the stretched structures.
Real-world applications may require alternative definitions of stretching intensity (e.g., based on percentage of maximum range of motion).

## Introduction

Exercise is widely recommended for health promotion [1, 2] but its effects critically depend on several loading variables (e.g., intensity, duration, volume, weekly frequency) [2–5]. While acknowledging their relevance and non-linear interactions in the definition of exercise *load*, the focus of this Current Opinion is on *intensity*. Different exercise intensities may produce distinct adaptations [6–8]. For example, in resistance training (RT), intensity is commonly defined in relation to a 1-repetition maximum (1-RM) test (e.g., 30% 1-RM, 85% 1-RM [9–11]) or maximum voluntary isometric contraction force (MVC), and greater strength gains are thought to be obtained through training at higher intensities [12], although there is some debate about this [13]. While 1-RM and MVC testing are common, other means to assess intensity in RT have been proposed (e.g., multiple RM [14, 15], minimum velocity threshold [9]).

For stretching, a very popular exercise modality [16, 17], intensity assessments are not so straightforward [18], and the clinical role of intensity is debatable [6, 18–20]. Stretching (in its various forms and flavors) is widely used for many stated purposes, with a range from acute post-exercise recovery [21] to long-term improvements in range of motion (ROM) [19], among several others. Despite its wide appeal and vast body of research (e.g., over 300 peer-reviewed empirical papers on competitive athletes alone [22]), the topic of defining and assessing stretching intensity is contentious. Stretch duration is commonly reported in seconds or minutes [23, 24], stretch volume in number of sets and repetitions or seconds (if a single set of static stretching is implemented) [25–27], and stretch frequency in the number of daily and/or weekly sessions [28, 29]. Conversely, reporting of stretch intensity is highly heterogeneous. This may partly explain why the role of stretching intensity in acute and chronic outcomes has yielded mixed results in the literature [19, 20, 30–32].

A recent large-scale scoping review [22] highlighted problems with the reporting of stretching intensity in research, including: (i) lack of any description; (ii) inter-study variation in outcome denoting the endpoint (e.g., pain^1^ vs discomfort); and (iii) inter-study variation in threshold for the endpoint (e.g., to point of initial discomfort, maximal discomfort, beyond the point of discomfort). A meta-analysis indicated that long-term structural adaptations to static stretching (e.g., increased fascicle length, muscle thickness) depend on stretching intensity when the assessment is performed via perceived rating of pain or discomfort [6]. However, while one systematic review showed that higher intensity static stretching training (defined as above the points of discomfort or pain) generated larger ROM gains [7], two other meta-analyses reported no significant differences in ROM with either high- or low-intensity stretching following an acute bout [20] or chronic stretch training [19]. Nonetheless, the question should be asked: can perceived pain or discomfort be considered the most accurate indicators of static stretching intensity? Pain and discomfort may only appear at greater magnitudes of stretching intensity (indeed, pain may not be experienced at all) and neither may be useful when the desired range falls well below the endpoint. Furthermore, some stretches are anatomically constrained, thereby precluding the onset of pain or discomfort (e.g., elbow extension ends upon bone-to-bone contact).

Objectively assessing the effectiveness of exercise training interventions with aims to optimize health, injury prevention, and rehabilitation [34, 35], and/or athletic performance [36, 37], relies on standardized the reporting of interventions [38–40] that enable an accurate contextualization of the findings and provide a solid basis for study replication [41, 42]. As load parameters, including intensity, influence specific adaptations to a training program [2], it is essential to understand dose–response relationships to properly prescribe an exercise intervention for targeted practical applications [3]. While in other interventions, such as strength and endurance training, intensities play a crucial role for physiological adaptations and are therefore clearly defined and applied in practice. Given the popularity of stretching as a training method [16, 17] without objective intensity prescriptions, it is timely to critically appraise how to define and assess stretching intensity [22]. Our goal is to review current methods for assessing static stretching intensity, including potential advantages and disadvantages, and to propose potential alternatives for further exploration. The aim is to raise awareness regarding the relevance and complexity of this topic, helping researchers to engage in a much needed discussion, while at the same time helping practitioners to assess available options and ponder their advantages and disadvantages.

The application of stretching intensity into exercise prescription is a distinct area of inquiry that would depend on specific training objectives (e.g., acute vs chronic adaptation, maximum ROM gains vs tissue stiffness reduction vs physical and mental relaxation). This aspect will not be addressed in this article.

## Perception-Based Methods for Assessing Stretching Intensity

Stretching intensity assessment is an acute problem, i.e., the aim is to quantify the intensity of a specific stretch, at a specific moment. Therefore, stretching intensity can be understood as *a mechanism-agnostic procedure*, in parallel with tests such as 1-RM and VO_2_max. How those data are used to prescribe specific exercise intensities is another problem and requires knowledge of the mechanisms driving the desired adaptations [4, 5]. Within this framework, reviews have shown that perception-based measurements (e.g., pain, discomfort) of static stretching intensity are common [22], and are especially predominant for static stretching protocols [6]. Even when included studies assess pre- and post-intervention ROM, the prescription is not usually based on the desired degrees of ROM or percentage of maximal ROM but on subjective perceptions of pain, discomfort, or other constructs [6, 22].

### Are Perception-Based Methods Truly Assessing Stretching Intensity?

In one meta-analysis [6], 18 of 19 studies using static stretching adopted perception-based assessments of stretching intensity. This highlights a fundamental and evident issue: although ROM tests quantify degrees or distances, participants are instructed to perform stretching at specific subjective intensities with no reference to such degrees or distances. This contrasts with RT, where a common practice in research is to prescribe intensity based on a percentage of a maximal lifted load or force produced (e.g., 1-RM, MVC).

A second problem is that the correlation between perception-based methods (e.g., visual analog scales commonly used to assess perceived pain [43]) and objective assessments is less than ideal and may be especially fragile for lower intensity loads, as was shown for RT [44]. For static stretching, intensity as measured through dynamometry was not significantly correlated with subjective pain perception assessed through a visual analog scale [45]. Another study used PNF (proprioceptive neuromuscular facilitation) stretching (which is admittedly distinct from static stretching) and determined intensity as a percentage of MVC [46]. The authors found a significant correlation between *post-*stretch pain (but not *during* stretching) and stretching intensity, but no correlation between perceived pain and ROM gains.

Pain perception varies considerably between individuals and may depend on several factors including pain history of the participants, previous experiences, and sex [47, 48]. Because exercise modulates pain perception [49, 50], it becomes relevant to consider whether a study was performed with previously untrained or trained participants. Attention, expectations, and reappraisal modulate pain perception [51], which may explain why the specific wording that is provided to participants before stretching influences the maximal ROM [52]. An experienced team of researchers observed that participants reached their maximal ROM (defined by perceived pain) earlier when visually monitoring the stretched joint. Consequently, they performed studies in which participants stretched with their eyes closed to eliminate visual feedback of the stretch amplitude [53, 54].

Pain perception also varies greatly as the body position, or position of non-stretched joints, varies. For example, pain may be strongly felt in the popliteal region (behind the knee) as the ankle is dorsiflexed to stretch the calf muscles when the hip joint is flexed but may be minimal or absent when the hip joint is extended during the stretch. This is at least partly due to the stretching of the tibial nerve during dorsiflexion when the hip is flexed, which is absent when the hip is extended [55, 56], and significantly reduces maximal ankle dorsiflexion ROM [57, 58]. Some researchers therefore perform calf muscle stretches (moving the ankle into dorsiflexion) with subjects at least partly reclined to minimize this pain [54, 59]. In such cases, pain perception itself would be of minimal use as an intensity marker.

As previously alluded to, some stretches may not even allow the participants to reach discomfort or pain. Elbow extension, for example, will be naturally bound by bone-on-bone contact and, depending on normal inter-individual variability, it may not allow everybody to reach discomfort or pain when performing a stretching of the region. For biarticular muscles, stretching amplitude might be increased by altering the position of other joints (e.g., shoulder extension to increase biceps brachii excursion). These arguments converge to suggest that perceived pain or discomfort may not provide an accurate measure of stretching intensity. Moreover, stretch tolerance responses may differ between men and women [60], and may even explain part of the differences in lower hamstring extensibility [61]. Therefore, using perception-based measures for prescribing stretching intensity may be population specific. So, while the constructs discussed in this section could be accepted as reasonably accurate proxies of stretching intensity, there are additional issues to consider.

### Inconsistency of Outcomes, Thresholds, and Terminology

Pain and discomfort tend to be used somewhat interchangeably in stretching research and their data might be pooled as a measure of stretching intensity [6], but they are not synonymous and will likely be interpreted differently by the participants [62, 63]. This affects inter-study comparability and the replication of results as some studies refer to the term “pain” [53, 64, 65], while others use the term “discomfort” [66, 67]. Moreover some studies use both interchangeably (e.g., “without discomfort or pain”), as recently underlined in two reviews [6, 22]. Some studies use alternative terms that are not synonyms of pain or discomfort, including “tolerable” or “feeling of stretch” [6, 22]. To compound the problem, even when two studies refer to the same outcome or terminology (e.g., pain), they do not necessarily use the same threshold (e.g., “without feeling pain” vs “onset of pain”), or the same terminology for what may be assumed as a comparable threshold (e.g., “without feeling pain” vs “preceding pain threshold”) [6, 22].

Regardless of exceptions whereby pain perception is compromised (e.g., congenital nociceptor deficiency [33], neuropathic pain [68]), a purported adaptation to stretching is increased stretch tolerance [69–72], even in the absence of measurable changes in muscle passive stiffness [73] or ROM [71]. The diffuse noxious inhibitory control theory of pain suggests that global reductions in pain sensitivity are modulated by the release of endorphins and enkephalins, which would alter pain perception with each repetition or set of stretching [20, 69]. This suggests that an identical stretch amplitude may be perceived differently depending on individual factors. Therefore, using pain, discomfort, or other similar outcomes likely does not provide an easily comparable measure of stretching intensity. Consistent with this, Lim and Park [45] found no rank correlation between perceived pain during stretching and the passive resistive torque produced.

### The Stretching Intensity Scale

The Stretching Intensity Scale [74] is an alternative perception-based method that assesses the perceived magnitude of stretching intensity. Participants are tested to experience maximal intensity stretching (i.e., maximal ROM) and then learn to report their perceived stretching intensity in relation to that threshold, with a range from none to supramaximal (the technical reason for including supramaximal intensity is addressed in Sect. 3.1). Despite being a subjective method, its specific language (i.e., directly related to the perception of stretching intensity) and its strong correlations with objective measures (e.g., ROM, passive torque) [74] provide a practical and potentially robust tool to consider in regular training applications. However, we are not aware of subsequent studies that could either support or question the validity and reliability of the Stretching Intensity Scale.

### Statistical Concerns When Analyzing Perception-Based Methods

The above methods use ordinal scales, where the intervals between the actual distances between points are neither equal nor empirically determined [75]. In practice, using the arbitrary example of an ordinal scale that ranges from 0 to 10, the difference between 5 and 6 will likely be of a different magnitude than the difference between 9 and 10. Numbers in an ordinal scale only provide a measure of order, not of interval [76], and research should avoid treating these data as if they represented a continuous set of values [75, 77–80]. This problem affects research on stretching intensity, including within meta-analyses [6, 19]. At a minimum, ordinal data should first be rescaled to an interval scale [80], and for pain scales it has been suggested that 95% confidence intervals should be used instead of the *p *value for interpreting the results [81]. The common practice of analyzing these scales through means and standard deviations assumes that the intervals between numbers are proportional (i.e., they require interval scales), which is untrue and may falsely suggest a proportional course of stretching pain and stretching intensity, which may not hold up to scrutiny [45].

Another problem arises when meta-analyses combine data from different studies to produce statements about the effects of stretching intensity [6]. Pooling data from studies using different outcomes (e.g., pain vs discomfort), inconsistent thresholds, and heterogeneous terminology for similar outcomes and thresholds poses a significant risk of providing misleading conclusions in meta-analyses. Unclear definitions of pain and discomfort, different definitions across studies, and complex relationships between subjective assessments and objective stretching effects contribute to substantial clinical heterogeneity between studies and preclude meta-analytical treatment [82, 83]. Meta-analyses that pool heterogeneous data are likely providing biased and invalid conclusions.

## Alternatives for Assessing Static Stretching Intensity

Perception-based methods to determine static stretching intensity are easy to apply in most settings, requiring little-to-no time and financial or human resource investment, but present important limitations that were previously discussed. Researchers and practitioners might find inspiration in the RT literature to implement objective methods to assess maximal intensity.^2^

### Percentage of Maximal Active ROM

Static stretching intensity can be determined based on maximal ROM [89]. To parallel common RT assessments, this would be the maximal *active* ROM, or *self-*ROM, without external aids. Participants would be asked to reach maximum ROM, i.e., where they cannot actively reach further. This maximal ROM would be assessed using goniometry (e.g., degree of hip flexion) or, in some cases, excursion distance (e.g., sit-and-reach), and would represent 100% intensity. Stretching intensity ranges would then be determined in relation to maximal active ROM [74].^3^ Importantly, the specific verbal instructions provided to achieve maximal ROM in terms of intensity (minimum, point, maximum) and sensation (tolerance, discomfort, pain) may change the maximal ROM and should be carefully considered and standardized [52].

Assessments should be joint and position specific, although we currently lack solid evidence to understand how position may change stretching intensity [18]. For example, the sit-and-reach and the stand-and-reach (also known as toe touch) tests are similar movements and involve the same joints; however, differences in body position and the resulting effects of gravity lead to relevant differences between the two, indicating that testing protocols should be specific. Moreover, the toe-touch test may be influenced by the sense of balance [90], which is unlikely to affect the sit-and-reach test.

Nonetheless, accurately measuring ROM requires (in most scenarios) an outside observer and this may not be practical (or feasible). Furthermore, torque or force during stretch varies non-linearly with ROM or distance, so the tissue load (stress) will vary markedly with only small changes in the joint angle or distance near to the maximum but vary less when stretches are performed further from it; i.e., changes in tissue stress or sensory load (pain, discomfort, pressure) may be minimal when moving from about 20 to 70% of maximum ROM or distance, but become substantial when advancing from 70 to 90% or from 90 to 100%. Finally, the ‘start of stretch’ can be hard to define, so subsequent calculations of percent ROM or distance may be difficult. Regardless, because the Stretching Intensity Scale presents large correlations with ROM [74], it may prove to be a useful proxy for a more objective measure.

### Maximal Tolerable Torque of the First Stretching Repetition

Research has also assessed and prescribed static stretching intensity as the angle (or distance) achieved at a given percentage of the peak passive torque during maximal stretch [8, 53, 54, 91–93], e.g., the ankle angle at which 90% of peak passive torque was reached [94]. Passive torque is determined at prescribed joint angles and passive resistive torque increases near the end ROM. As maximal ROM varies between individuals, and joint anatomical properties might influence maximal ROM, it is possible to move to the angle at which a given percentage of maximal passive torque occurs to achieve a similar stretch intensity. The passive torque decreases within a stretching session owing to relaxation effects [95] or holding the stretch for a short period of time [96], consistent with non-linear increases observed in ROM in successive repetitions (hysteresis) [97]. The end-ROM intensity could become more objective after completion of an appropriate task-specific warm-up. When stretch intensity is high and passive torque (or resistive force) decreases via the stress relaxation response, the joint angle or stretch distance can be increased to maintain the target passive torque level, allowing for real-time changes to maintain intensity as biomechanical and physiological adaptations occur, for example, as in ‘constant torque’ stretching [96, 98–100].

### Muscle Stiffness

Stiffness refers to the amount of force or stress opposing tissue deformation, and muscle stiffness plays a pivotal role in determining maximal ROM [101], although it may vary depending on the joint and specific movement [102]. Conversely, tendon stiffness appears to have little influence on ROM [53, 103], although its effects on perceived stretch, discomfort, or pain should be explored in future research. Strong linear relationships have been demonstrated between muscle stiffness and the passive muscle force during the passive stretch [104–107]. Therefore, muscle stiffness could provide an objective approach to determine stretching intensity, as stiffness would increase with increasing muscle length. Stiffness may vary depending on joint position (e.g., neutral vs flexed) [108, 109]. However, muscle tissues are not the sole determinants of ROM, and indeed their relative importance in limiting ROM may decrease with age [110].

**Fig. 1:**
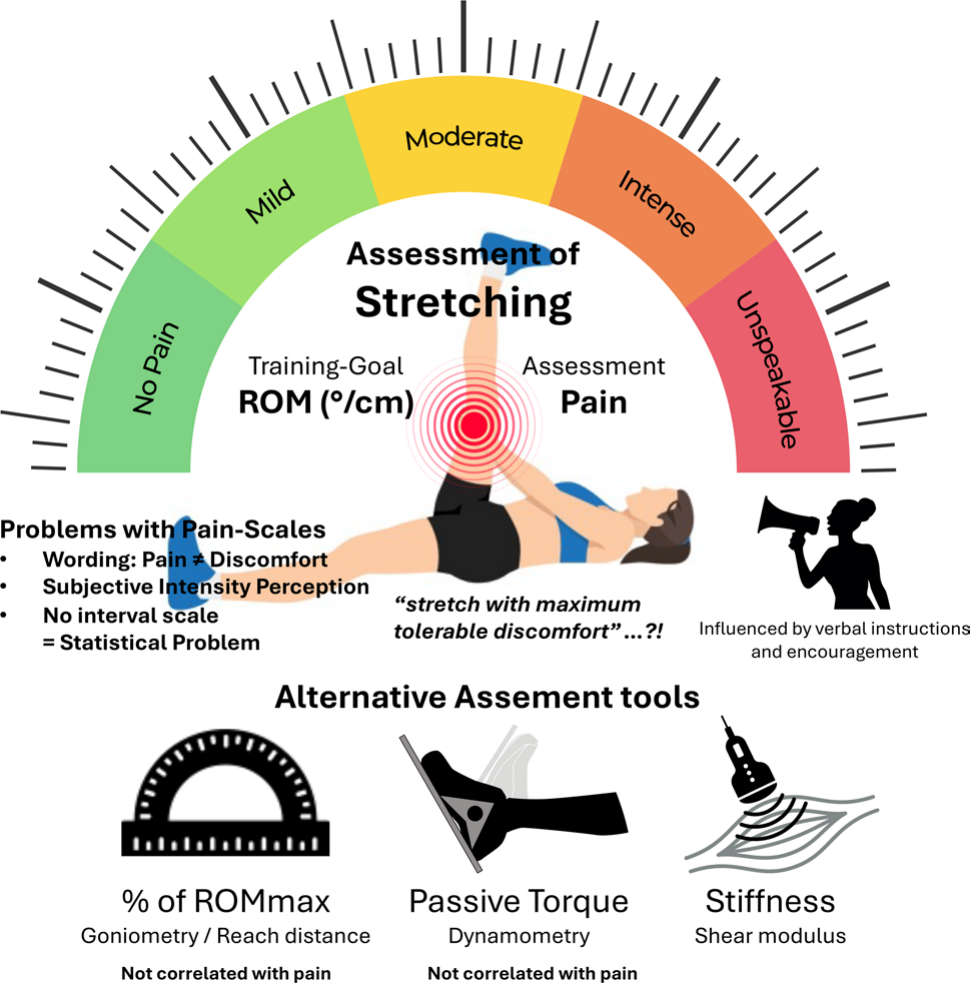
Different methods to determine stretch intensity. *ROM* range of motion, *ROMmax* maximal ROM

Given the complexities in the set-up to assess stiffness, this method may not be feasible for ongoing applications typical of “real-world” training settings, although it may be useful for scientific research. As for passive torque, stiffness assessments are meaningful only if associated with maximal ROM. In cases where other structures (e.g., nerve, bone, joint capsule) greatly influence ROM (e.g., for some joints or movements), stiffness may become a less useful parameter (e.g., as previously mentioned in the study by Reiner et al. [102]). In addition, because the muscle force–length relationship generally mirrors the joint torque–angle relationship, peak passive torque might be a convenient proxy for the muscle stiffness assessment. Figure 1 synthesizes the methods discussed in this Current Opinion.

## Conclusions

Stretch intensity is an important load control parameter influencing stretching outcomes but lacks a consensual definition. Current conclusions and recommendations regarding the implementation of different intensities lack validity because of unclear definitions of stretching intensity and the absence of standardized assessment procedures. Moreover, the wide variation in assessment methods renders inter-study comparisons difficult. The use of perception-based scales, while practical, has several limitations that curb its scientific validity. Although it is theoretically sound to use parameters such as passive torque or force or muscle stiffness, these approaches remain unvalidated and future studies are needed before investing further in testing approaches with higher practical or clinical relevance. For researchers, we call for further discussion on the topic; for practitioners, we suggest the adoption of practical measures to assess stretching intensity while avoiding definitive statements on the topic.

## Footnotes

1 Pain and nociception are different constructs, but are often (and misguidedly) conflated [33]. An exploration of this issue would stray far from the scope of this article, and therefore we use the term “pain” as commonly used in the stretching literature [6, 22].

2 Assessments may vary depending on ambient temperature, time of day, and other fluctuating factors, which is why standardization of test conditions is so important [2, 84, 85]. Likewise, a familiarization period is necessary, or otherwise changes may result exclusively from learning the test instead of reflecting “true” outcomes [86–88]. Periodic assessments are required to update the reference values.

3 Acute stretch tolerance may improve ROM in subsequent repetitions, and it is therefore possible to talk of “supramaximal ROM” [74]. Two options are available: (i) assess a single repetition and consider the possibility of supramaximal percentages; or (ii) assess multiple repetitions and consider the best attempt to reflect “true” maximal intensity, whereby supramaximal percentages would only be applicable to assisted stretches.

## References

[1] Bull FC, Al-Ansari SS, Biddle S, Borodulin K, Buman MP, Cardon G, et al. World Health Organization 2020 guidelines on physical activity and sedentary behaviour. Br J Sports Med. 2020;54(24):1451–62. 10.1136/bjsports-2020-102955. 33239350 10.1136/bjsports-2020-102955 PMC7719906

[2] ACSM, Liguori G, Feito Y, Fountaine C, Roy BA. ACSM’s guidelines for exercise testing and prescription. 11th ed. Philadelphia: Wolters Kluwer; 2022.

[3] Gronwald T, Törpel A, Herold F, Budde H. Perspective of dose and response for individualized physical exercise and training prescription. J Funct Morphol Kinesiol. 2020;5(3):48. 10.3390/jfmk5030048. 33467264 10.3390/jfmk5030048 PMC7739365

[4] Impellizzeri FM, Marcora SM, Coutts AJ. Internal and external training load: 15 years on. Int J Sports Physiol Perform. 2019;14(2):270–3. 10.1123/ijspp.2018-0935. 30614348 10.1123/ijspp.2018-0935

[5] Kalkhoven JT, Watsford ML, Coutts AJ, Edwards WB, Impellizzeri FM. Training load and injury: causal pathways and future directions. Sports Med. 2021;51(6):1137–50. 10.1007/s40279-020-01413-6. 33400216 10.1007/s40279-020-01413-6

[6] Panidi I, Donti O, Konrad A, Dinas PC, Terzis G, Mouratidis A, et al. Muscle architecture adaptations to static stretching training: a systematic review with meta-analysis. Sports Med Open. 2023;9(1):47. 10.1186/s40798-023-00591-7. 37318696 10.1186/s40798-023-00591-7 PMC10271914

[7] Bryant J, Cooper DJ, Peters DM, Cook MD. The effects of static stretching intensity on range of motion and strength: a systematic review. J Funct Morphol Kinesiol. 2023;8(2):37. 10.3390/jfmk8020037. 37092369 10.3390/jfmk8020037 PMC10123604

[8] Freitas SR, Vilarinho D, Rocha Vaz J, Bruno PM, Costa PB, Mil-homens P. Responses to static stretching are dependent on stretch intensity and duration. Clin Physiol Funct Imaging. 2015;35(6):478–84. 10.1111/cpf.12186. 25164268 10.1111/cpf.12186

[9] LeMense AT, Malone GT, Kinderman MA, Fedewa MV, Winchester LJ. Validity of using the load-velocity relationship to estimate 1 repetition maximum in the back squat exercise: a systematic review and meta-analysis. J Strength Cond Res. 2024;38(3):612–9. 10.1519/jsc.0000000000004709. 38416447 10.1519/JSC.0000000000004709

[10] Grgic J, Lazinica B, Schoenfeld BJ, Pedisic Z. Test-retest reliability of the one-repetition maximum (1RM) strength assessment: a systematic review. Sports Med Open. 2020;6(1):31. 10.1186/s40798-020-00260-z. 32681399 10.1186/s40798-020-00260-z PMC7367986

[11] Warneke K, Wagner CM, Keiner M, Hillebrecht M, Schiemann S, Behm DG, et al. Maximal strength measurement: a critical evaluation of common methods: a narrative review. Front Sports Act Living. 2023;5:Article 1105201. 10.3389/fspor.2023.1105201. 36873661 10.3389/fspor.2023.1105201 PMC9981657

[12] Lopez P, Radaelli R, Taaffe DR, Newton RU, Galvão DA, Trajano GS, et al. Resistance training load effects on muscle hypertrophy and strength gain: systematic review and network meta-analysis. Med Sci Sports Exerc. 2021;53(6):1206–16. 10.1249/mss.0000000000002585. 33433148 10.1249/MSS.0000000000002585 PMC8126497

[13] Schoenfeld BJ, Grgic J, Van Every DW, Plotkin DL. Loading recommendations for muscle strength, hypertrophy, and local endurance: a re-examination of the repetition continuum. Sports (Basel). 2021;9(2):32. 10.3390/sports9020032. 33671664 10.3390/sports9020032 PMC7927075

[14] Gail S, Künzell S. Reliability of a 5-repetition maximum strength test in recreational athletes. Dtsch Z Sportmed. 2014;65(11):314–7. 10.5960/dzsm.2014.138.

[15] Reynolds JM, Gordon TJ, Robergs RA. Prediction of one repetition maximum strength from multiple repetition maximum testing and anthropometry. J Strength Cond Res. 2006;20(3):584–92. 10.1519/r-15304.1. 16937972 10.1519/R-15304.1

[16] Popp JK, Bellar DM, Hoover DL, Craig BW, Leitzelar BN, Wanless EA, et al. Pre- and post-activity stretching practices of collegiate athletic trainers in the United States. J Strength Cond Res. 2017;31(9):2347–54. 10.1519/JSC.0000000000000890. 25734784 10.1519/JSC.0000000000000890

[17] Babault N, Rodot G, Champelovier M, Cometti C. A survey on stretching practices in women and men from various sports or physical activity programs. Int J Environ Res Public Health. 2021;18(8):3928. 10.3390/ijerph18083928. 33918033 10.3390/ijerph18083928 PMC8068839

[18] Apostolopoulos N, Metsios GS, Flouris AD, Koutedakis Y, Wyon MA. The relevance of stretch intensity and position—a systematic review. Front Psychol. 2015;6:1128. 10.3389/fpsyg.2015.01128. 26347668 10.3389/fpsyg.2015.01128 PMC4540085

[19] Konrad A, Alizadeh S, Daneshjoo A, Anvar SH, Graham A, Zahiri A, et al. Chronic effects of stretching on range of motion with consideration of potential moderating variables: a systematic review with meta-analysis. J Sport Health Sci. 2024;13(2):186–94. 10.1016/j.jshs.2023.06.002. 37301370 10.1016/j.jshs.2023.06.002 PMC10980866

[20] Behm DG, Alizadeh S, Daneshjoo A, Anvar SH, Graham A, Zahiri A, et al. Acute effects of various stretching techniques on range of motion: a systematic review with meta-analysis. Sports Med Open. 2023;9(1):107. 10.1186/s40798-023-00652-x. 37962709 10.1186/s40798-023-00652-x PMC10645614

[21] Afonso J, Clemente FM, Nakamura FY, Morouço P, Sarmento H, Inman RA, et al. The effectiveness of post-exercise stretching in short-term and delayed recovery of strength, range of motion and delayed onset muscle soreness: a systematic review and meta-analysis of randomized controlled trials. Front Physiol. 2021;12:Article 677581. 10.3389/fphys.2021.677581. 34025459 10.3389/fphys.2021.677581 PMC8133317

[22] Afonso J, Andrade R, Rocha-Rodrigues S, Nakamura FY, Sarmento H, Freitas SR, et al. What we do not know about stretching in healthy athletes: a scoping review with evidence gap map from 300 trials. Sports Med. 2024;54(6):1517–51. 10.1007/s40279-024-02002-7. 38457105 10.1007/s40279-024-02002-7 PMC11239752

[23] Moustafa IM, Ahbouch A, Palakkottuparambil F, Walton LM. Optimal duration of stretching of the hamstring muscle group in older adults: a randomized controlled trial. Eur J Phys Rehabil Med. 2021;57(6):931–9. 10.23736/s1973-9087.21.06731-9. 34002974 10.23736/S1973-9087.21.06731-9

[24] Warneke K, Wirth K, Keiner M, Schiemann S. Improvements in flexibility depend on stretching duration. Int J Exerc Sci. 2023;16(4):83–94. 37113511 10.70252/LBOU2008 PMC10124737

[25] Yahata K, Konrad A, Sato S, Kiyono R, Yoshida R, Fukaya T, et al. Effects of a high-volume static stretching programme on plantar-flexor muscle strength and architecture. Eur J Appl Physiol. 2021;121(4):1159–66. 10.1007/s00421-021-04608-5. 33502614 10.1007/s00421-021-04608-5

[26] Lima TP, Farinatti PT, Rubini EC, Silva EB, Monteiro WD. Hemodynamic responses during and after multiple sets of stretching exercises performed with and without the Valsalva maneuver. Clinics. 2015;70(5):333–8. 10.6061/clinics/2015(05)05. 26039949 10.6061/clinics/2015(05)05 PMC4449462

[27] Johnson AW, Mitchell UH, Meek K, Feland JB. Hamstring flexibility increases the same with 3 or 9 repetitions of stretching held for a total time of 90 s. Phys Ther Sport. 2014;15(2):101–5. 10.1016/j.ptsp.2013.03.006. 23896197 10.1016/j.ptsp.2013.03.006

[28] Webber SC, Porter MM. Effect of a supervised stretching program on neck, shoulder, and trunk range of motion in older women. Can J Aging. 2022;41(3):297–303. 10.1017/s0714980821000350. 35859360 10.1017/S0714980821000350

[29] Mason JS, Crowell M, Dolbeer J, Morris J, Terry A, Koppenhaver S, et al. The effectiveness of dry needling and stretching vs. stretching alone on hamstring flexibility in patients with knee pain: a randomized controlled trial. Int J Sports Phys Ther. 2016;11(5):672–83. 27757280 PMC5046961

[30] Fukaya T, Sato S, Yahata K, Yoshida R, Takeuchi K, Nakamura M. Effects of stretching intensity on range of motion and muscle stiffness: a narrative review. J Bodyw Mov Ther. 2022;32:68–76. 10.1016/j.jbmt.2022.04.011. 36180161 10.1016/j.jbmt.2022.04.011

[31] Fukaya T, Matsuo S, Iwata M, Yamanaka E, Tsuchida W, Asai Y, et al. Acute and chronic effects of static stretching at 100% versus 120% intensity on flexibility. Eur J Appl Physiol. 2021;121(2):513–23. 10.1007/s00421-020-04539-7. 33151438 10.1007/s00421-020-04539-7

[32] Nakamura M, Yoshida R, Sato S, Yahata K, Murakami Y, Kasahara K, et al. Cross-education effect of 4-week high- or low-intensity static stretching intervention programs on passive properties of plantar flexors. J Biomech. 2022;133:Article 110958. 10.1016/j.jbiomech.2022.110958. 35078021 10.1016/j.jbiomech.2022.110958

[33] Weisman A, Quintner J, Masharawi Y. Congenital insensitivity to pain: a misnomer. J Pain. 2019;20(9):1011–4. 10.1016/j.jpain.2019.01.331. 30716471 10.1016/j.jpain.2019.01.331

[34] McBain K, Shrier I, Shultz R, Meeuwisse WH, Klügl M, Garza D, et al. Prevention of sports injury I: a systematic review of applied biomechanics and physiology outcomes research. Br J Sports Med. 2012;46(3):169–73. 10.1136/bjsm.2010.080929. 21508076 10.1136/bjsm.2010.080929

[35] McBain K, Shrier I, Shultz R, Meeuwisse WH, Klügl M, Garza D, et al. Prevention of sport injury part II: a systematic review of clinical science research. Br J Sports Med. 2012;46(3):174–9. 10.1136/bjsm.2010.081182. 21471144 10.1136/bjsm.2010.081182

[36] McLean S, Kerhervé HA, Stevens N, Salmon PM. A systems analysis critique of sport-science research. Int J Sports Physiol Perform. 2021;16(10):1385–92. 10.1123/ijspp.2020-0934. 34453014 10.1123/ijspp.2020-0934

[37] Crowley E, Harrison AJ, Lyons M. The impact of resistance training on swimming performance: a systematic review. Sports Med. 2017;47(11):2285–307. 10.1007/s40279-017-0730-2. 28497283 10.1007/s40279-017-0730-2

[38] Verhagen E, Clarsen B, Capel-Davies J, Collins C, Derman W, de Winter D, et al. Tennis-specific extension of the International Olympic Committee consensus statement: methods for recording and reporting of epidemiological data on injury and illness in sport 2020. Br J Sports Med. 2021;55(1):9–13. 10.1136/bjsports-2020-102360. 33082146 10.1136/bjsports-2020-102360 PMC7788227

[39] Slade SC, Dionne CE, Underwood M, Buchbinder R, Beck B, Bennell K, et al. Consensus on exercise reporting template (CERT): modified delphi study. Phys Ther. 2016;96(10):1514–24. 10.2522/ptj.20150668. 27149962 10.2522/ptj.20150668

[40] Slade SC, Keating JL. Exercise prescription: a case for standardised reporting. Br J Sports Med. 2012;46(16):1110–3. 10.1136/bjsports-2011-090290. 22089077 10.1136/bjsports-2011-090290

[41] Ekkekakis P, Swinton P, Tiller NB. Extraordinary claims in the literature on high-intensity interval training (HIIT): I. bonafide scientific revolution or a looming crisis of replication and credibility? Sports Med. 2023;53(10):1865–90. 10.1007/s40279-023-01880-7. 37561389 10.1007/s40279-023-01880-7

[42] Murphy J, Mesquida C, Caldwell AR, Earp BD, Warne JP. Proposal of a selection protocol for replication of studies in sports and exercise science. Sports Med. 2023;53(1):281–91. 10.1007/s40279-022-01749-1. 36066754 10.1007/s40279-022-01749-1 PMC9807474

[43] Åström M, Thet Lwin ZM, Teni FS, Burström K, Berg J. Use of the visual analogue scale for health state valuation: a scoping review. Qual Life Res. 2023;32(10):2719–29. 10.1007/s11136-023-03411-3. 37029258 10.1007/s11136-023-03411-3 PMC10474194

[44] Leung AWS, Chan CCH, Lee AHS, Lam KWH. Visual analogue scale correlates of musculoskeletal fatigue. Percept Mot Skills. 2004;99(1):235–46. 10.2466/pms.99.1.235-246. 15446651 10.2466/pms.99.1.235-246

[45] Lim W, Park H. No significant correlation between the intensity of static stretching and subject’s perception of pain. J Phys Ther Sci. 2017;29(10):1856–9. 10.1589/jpts.29.1856. 29184306 10.1589/jpts.29.1856 PMC5684027

[46] Lim W. Optimal intensity of PNF stretching: maintaining the efficacy of stretching while ensuring its safety. J Phys Ther Sci. 2018;30(8):1108–11. 10.1589/jpts.30.1108. 30154610 10.1589/jpts.30.1108 PMC6110207

[47] Fillingim RB. Individual differences in pain: understanding the mosaic that makes pain personal. Pain. 2017;158(Suppl. 1):S11–8. 10.1097/j.pain.0000000000000775. 27902569 10.1097/j.pain.0000000000000775 PMC5350021

[48] Coghill RC. Individual differences in the subjective experience of pain: new insights into mechanisms and models. Headache. 2010;50(9):1531–5. 10.1111/j.1526-4610.2010.01763.x. 20958300 10.1111/j.1526-4610.2010.01763.x PMC2959190

[49] Patti A, Bianco A, Karsten B, Montalto MA, Battaglia G, Bellafiore M, et al. The effects of physical training without equipment on pain perception and balance in the elderly: a randomized controlled trial. Work (Reading, Mass). 2017;57(1):23–30. 10.3233/wor-172539. 28506013 10.3233/WOR-172539 PMC5467714

[50] Pettersen SD, Aslaksen PM, Pettersen SA. Pain processing in elite and high-level athletes compared to non-athletes. Front Psychol. 2020;11:1908. 10.3389/fpsyg.2020.01908. 32849117 10.3389/fpsyg.2020.01908 PMC7399202

[51] Wiech K, Ploner M, Tracey I. Neurocognitive aspects of pain perception. Trends Cogn Sci. 2008;12(8):306–13. 10.1016/j.tics.2008.05.005. 18606561 10.1016/j.tics.2008.05.005

[52] Radaelli R, Freitas J, Almeida N, Vaz JR, Freitas SR. Which stretching instruction should be given to assess joint maximal range of motion? J Bodyw Mov Ther. 2022;31:45–50. 10.1016/j.jbmt.2022.04.010. 35710220 10.1016/j.jbmt.2022.04.010

[53] Blazevich AJ, Cannavan D, Waugh CM, Miller SC, Thorlund JB, Aagaard P, et al. Range of motion, neuromechanical, and architectural adaptations to plantar flexor stretch training in humans. J Appl Physiol. 2014;117(5):452–62. 10.1152/japplphysiol.00204.2014. 24947023 10.1152/japplphysiol.00204.2014

[54] Blazevich AJ, Cannavan D, Waugh CM, Fath F, Miller SC, Kay AD. Neuromuscular factors influencing the maximum stretch limit of the human plantar flexors. J Appl Physiol (1985). 2012;113(9):1446–55. 10.1152/japplphysiol.00882.2012. 22923509 10.1152/japplphysiol.00882.2012

[55] Coppieters MW, Alshami AM, Babri AS, Souvlis T, Kippers V, Hodges PW. Strain and excursion of the sciatic, tibial, and plantar nerves during a modified straight leg raising test. J Orthop Res. 2006;24(9):1883–9. 10.1002/jor.20210. 16838375 10.1002/jor.20210

[56] Shum GL, Attenborough AS, Marsden JF, Hough AD. Tibial nerve excursion during lumbar spine and hip flexion measured with diagnostic ultrasound. Ultrasound Med Biol. 2013;39(5):784–90. 10.1016/j.ultrasmedbio.2012.11.023. 23465136 10.1016/j.ultrasmedbio.2012.11.023

[57] Mitchell B, Bressel E, McNair PJ, Bressel ME. Effect of pelvic, hip, and knee position on ankle joint range of motion. Phys Ther Sport. 2008;9(4):202–8. 10.1016/j.ptsp.2008.08.002. 19083721 10.1016/j.ptsp.2008.08.002

[58] Andrade RJ, Lacourpaille L, Freitas SR, McNair PJ, Nordez A. Effects of hip and head position on ankle range of motion, ankle passive torque, and passive gastrocnemius tension. Scand J Med Sci Sports. 2016;26(1):41–7. 10.1111/sms.12406. 25676048 10.1111/sms.12406

[59] Cannavan D, Coleman DR, Blazevich AJ. Lack of effect of moderate-duration static stretching on plantar flexor force production and series compliance. Clin Biomech (Bristol, Avon). 2012;27(3):306–12. 10.1016/j.clinbiomech.2011.10.003. 10.1016/j.clinbiomech.2011.10.003 22047756

[60] Hoge KM, Ryan ED, Costa PB, Herda TJ, Walter AA, Stout JR, et al. Gender differences in musculotendinous stiffness and range of motion after an acute bout of stretching. J Strength Cond Res. 2010;24(10):2618–26. 10.1519/JSC.0b013e3181e73974. 20885189 10.1519/JSC.0b013e3181e73974

[61] Marshall PW, Siegler JC. Lower hamstring extensibility in men compared to women is explained by differences in stretch tolerance. BMC Musculoskelet Disord. 2014;15:223. 10.1186/1471-2474-15-223. 25000977 10.1186/1471-2474-15-223 PMC4105123

[62] Funabashi M, Wang S, Lee AD, Duarte FCK, Budgell B, Stilwell P, et al. Discomfort, pain and stiffness: what do these terms mean to patients? A cross-sectional survey with lexical and qualitative analyses. BMC Musculoskelet Disord. 2022;23(1):283. 10.1186/s12891-022-05214-y. 35331201 10.1186/s12891-022-05214-y PMC8944041

[63] Ashkenazy S, DeKeyser Ganz F. The differentiation between pain and discomfort: a concept analysis of discomfort. Pain Manag Nurs. 2019;20(6):556–62. 10.1016/j.pmn.2019.05.003. 31307870 10.1016/j.pmn.2019.05.003

[64] Kataura S, Suzuki S, Matsuo S, Hatano G, Iwata M, Yokoi K, et al. Acute effects of the different intensity of static stretching on flexibility and isometric muscle force. J Strength Cond Res. 2017;31(12):3403–10. 27984497 10.1519/JSC.0000000000001752

[65] Hatano G, Matsuo S, Asai Y, Suzuki S, Iwata M. Effects of high-intensity stretch with moderate pain and maximal intensity stretch without pain on flexibility. J Sports Sci Med. 2022;21(2):171–81. 10.52082/jssm.2022.171. 35719229 10.52082/jssm.2022.171 PMC9157514

[66] Freitas SR, Andrade RJ, Nordez A, Mendes B, Mil-Homens P. Acute muscle and joint mechanical responses following a high-intensity stretching protocol. Eur J Appl Physiol. 2016;116(8):1519–26. 10.1007/s00421-016-3410-2. 27270900 10.1007/s00421-016-3410-2

[67] Fukaya T, Kiyono R, Sato S, Yahata K, Yasaka K, Onuma R, et al. Effects of static stretching with high-intensity and short-duration or low-intensity and long-duration on range of motion and muscle stiffness. Front Physiol. 2020;11:601912. 10.3389/fphys.2020.601912. 33329054 10.3389/fphys.2020.601912 PMC7714915

[68] Finnerup NB, Kuner R, Jensen TS. Neuropathic pain: from mechanisms to treatment. Physiol Rev. 2021;101(1):259–301. 10.1152/physrev.00045.2019. 32584191 10.1152/physrev.00045.2019

[69] Behm DG, Blazevich AJ, Kay AD, McHugh M. Acute effects of muscle stretching on physical performance, range of motion, and injury incidence in healthy active individuals: a systematic review. Appl Physiol Nutr Metab. 2016;41(1):1–11. 10.1139/apnm-2015-0235. 26642915 10.1139/apnm-2015-0235

[70] Law RY, Harvey LA, Nicholas MK, Tonkin L, De Sousa M, Finniss DG. Stretch exercises increase tolerance to stretch in patients with chronic musculoskeletal pain: a randomized controlled trial. Phys Ther. 2009;89(10):1016–26. 10.2522/ptj.20090056. 19696119 10.2522/ptj.20090056

[71] Folpp H, Deall S, Harvey LA, Gwinn T. Can apparent increases in muscle extensibility with regular stretch be explained by changes in tolerance to stretch? Aust J Physiother. 2006;52(1):45–50. 10.1016/s0004-9514(06)70061-7. 16515422 10.1016/s0004-9514(06)70061-7

[72] Weppler CH, Magnusson SP. Increasing muscle extensibility: a matter of increasing length or modifying sensation? Phys Ther. 2010;90(3):438–49. 10.2522/ptj.20090012. 20075147 10.2522/ptj.20090012

[73] Muanjai P, Jones DA, Mickevicius M, Satkunskiene D, Snieckus A, Skurvydas A, et al. The acute benefits and risks of passive stretching to the point of pain. Eur J Appl Physiol. 2017;117(6):1217–26. 10.1007/s00421-017-3608-y. 28391391 10.1007/s00421-017-3608-y

[74] Freitas SR, Vaz JR, Gomes L, Silvestre R, Hilário E, Cordeiro N, et al. A new tool to assess the perception of stretching intensity. J Strength Cond Res. 2015;29(9):2666–78. 10.1519/jsc.0000000000000926. 25763516 10.1519/JSC.0000000000000926

[75] Grimby G, Tennant A, Tesio L. The use of raw scores from ordinal scales: time to end malpractice? J Rehabil Med. 2012;44(2):97–8. 10.2340/16501977-0938. 22334345 10.2340/16501977-0938

[76] Shukla D. A narrative review on types of data and scales of measurement: an initial step in the statistical analysis of medical data. Cancer Res Stat Treat. 2023;6(2):279–83.

[77] Kemp S, Grace RC. When can information from ordinal scale variables be integrated? Psychol Methods. 2010;15(4):398–412. 10.1037/a0021462. 21133543 10.1037/a0021462

[78] Stijic M, Messerer B, Meißner W, Avian A. Numeric rating scale for pain should be used in an ordinal but not interval manner: a retrospective analysis of 346,892 patient reports of the quality improvement in postoperative pain treatment registry. Pain. 2024;165(3):707–14. 10.1097/j.pain.0000000000003078. 37851363 10.1097/j.pain.0000000000003078

[79] Miot HA. Analysis of ordinal data in clinical and experimental studies. J Vasc Bras. 2020;19:e20200185. 10.1590/1677-5449.200185. 34211532 10.1590/1677-5449.200185 PMC8217997

[80] Harwell MR, Gatti GG. Rescaling ordinal data to interval data in educational research. Rev Educ Res. 2001;71(1):105–31. 10.3102/00346543071001105.

[81] Mantha S, Thisted R, Foss J, Ellis JE, Roizen MF. A proposal to use confidence intervals for visual analog scale data for pain measurement to determine clinical significance. Anesth Analg. 1993;77(5):1041–7. 8214704 10.1213/00000539-199311000-00029

[82] Lensen S. When to pool data in a meta-analysis (and when not to)? Fertil Steril. 2023;119(6):902–3. 10.1016/j.fertnstert.2023.03.015. 36948444 10.1016/j.fertnstert.2023.03.015

[83] Higgins JP, Thomas J, Chandler J, Cumpston M, Li T, Page MJ, et al. Cochrane handbook for systematic reviews of interventions. 2nd ed. Chichester: Wiley; 2019.

[84] Petrigna L, Pajaujiene S, Delextrat A, Gómez-López M, Paoli A, Palma A, et al. The importance of standard operating procedures in physical fitness assessment: a brief review. Sport Sci Health. 2022;18(1):21–6. 10.1007/s11332-021-00849-1.

[85] Fletcher GF, Balady GJ, Amsterdam EA, Chaitman B, Eckel R, Fleg J, et al. Exercise standards for testing and training. Circulation. 2001;104(14):1694–740. 10.1161/hc3901.095960. 11581152 10.1161/hc3901.095960

[86] Vrbik I, Sporiš G, Štefan L, Madić D, Trajković N, Valantine I, et al. The influence of familiarization on physical fitness test results in primary school-aged children. Pediatr Exerc Sci. 2017;29(2):278–84. 10.1123/pes.2016-0091. 27768554 10.1123/pes.2016-0091

[87] Drake D, Kennedy R, Wallace E. Familiarization, validity and smallest detectable difference of the isometric squat test in evaluating maximal strength. J Sports Sci. 2018;36(18):2087–95. 10.1080/02640414.2018.1436857. 29405842 10.1080/02640414.2018.1436857

[88] McGoldrick CD, Iacono AD, Morgan OJ, Nayler J, Buchanan J, McCart C, et al. Reliability, familiarization effect, and comparisons between a predetermined and a self-determined isometric-squat testing protocol. Int J Sports Physiol Perform. 2023;18(7):718–25. 10.1123/ijspp.2022-0480. 37207996 10.1123/ijspp.2022-0480

[89] Takeuchi K, Akizuki K, Nakamura M. Acute effects of different intensity and duration of static stretching on the muscle-tendon unit stiffness of the hamstrings. J Sports Sci Med. 2022;21(4):528–35. 10.52082/jssm.2022.528. 36523898 10.52082/jssm.2022.528 PMC9741716

[90] Siqueira CM, Rossi A, Shimamoto C, Tanaka C. Balance highly influences flexibility measured by the toe-touch test. Hum Mov Sci. 2018;62:116–23. 10.1016/j.humov.2018.10.001. 30300805 10.1016/j.humov.2018.10.001

[91] Freitas SR, Vaz JR, Bruno PM, Andrade R, Mil-Homens P. Stretching effects: high-intensity and moderate-duration vs. low-intensity and long-duration. Int J Sports Med. 2016;37(3):239–44. 10.1055/s-0035-1548946. 26701828 10.1055/s-0035-1548946

[92] Oba K, Samukawa M, Nakamura K, Mikami K, Suzumori Y, Ishida Y, et al. Influence of constant torque stretching at different stretching intensities on flexibility and mechanical properties of plantar flexors. J Strength Cond Res. 2021;35(3):709–14. 10.1519/JSC.0000000000002767. 30052602 10.1519/JSC.0000000000002767

[93] Lim W. Easy method for measuring stretching intensities in real clinical settings and effects of different stretching intensities on flexibility. J Back Musculoskelet Rehabil. 2019;32(4):579–85. 10.3233/bmr-181243. 30530964 10.3233/BMR-181243

[94] Blazevich AJ, Kay AD, Waugh C, Fath F, Miller S, Cannavan D. Plantarflexor stretch training increases reciprocal inhibition measured during voluntary dorsiflexion. J Neurophysiol. 2012;107(1):250–6. 10.1152/jn.00407.2011. 21975448 10.1152/jn.00407.2011

[95] Magnusson SP, Simonsen EB, Dyhre-Poulsen P, Aagaard P, Mohr T, Kjaer M. Viscoelastic stress relaxation during static stretch in human skeletal muscle in the absence of EMG activity. Scand J Med Sci Sports. 1996;6(6):323–8. 10.1111/j.1600-0838.1996.tb00101.x. 9046541 10.1111/j.1600-0838.1996.tb00101.x

[96] Wohlann T, Warneke K, Kalder V, Behm DG, Schmidt T, Schiemann S. Influence of 8-weeks of supervised static stretching or resistance training of pectoral major muscles on maximal strength, muscle thickness and range of motion. Eur J Appl Physiol. 2024;124(6):1885–93. 10.1007/s00421-023-05413-y. 38240811 10.1007/s00421-023-05413-y PMC11129965

[97] Holzgreve F, Maurer-Grubinger C, Isaak J, Kokott P, Mörl-Kreitschmann M, Polte L, et al. The acute effect in performing common range of motion tests in healthy young adults: a prospective study. Sci Rep. 2020;10(1):21722. 10.1038/s41598-020-78846-6. 33303934 10.1038/s41598-020-78846-6 PMC7728808

[98] Trajano GS, Seitz L, Nosaka K, Blazevich AJ. Contribution of central vs. peripheral factors to the force loss induced by passive stretch of the human plantar flexors. J Appl Physiol. 2013;115(2):212–8. 10.1152/japplphysiol.00333.2013. 23661620 10.1152/japplphysiol.00333.2013

[99] Yeh C-Y, Tsai K-H, Chen J-J. Effects of prolonged muscle stretching with constant torque or constant angle on hypertonic calf muscles. Arch Phys Med Rehabil. 2005;86(2):235–41. 10.1016/j.apmr.2004.03.032. 15706549 10.1016/j.apmr.2004.03.032

[100] Herda TJ, Costa PB, Walter AA, Ryan ED, Cramer JT. The time course of the effects of constant-angle and constant-torque stretching on the muscle–tendon unit. Scand J Med Sci Sports. 2014;24(1):62–7. 10.1111/j.1600-0838.2012.01492.x. 22738303 10.1111/j.1600-0838.2012.01492.x

[101] Hirata K, Akagi R. Contribution of muscle stiffness of the triceps surae to passive ankle joint stiffness in young and older adults. Front Physiol. 2022;13:Article 972755. 10.3389/fphys.2022.972755. 36726380 10.3389/fphys.2022.972755 PMC9885261

[102] Reiner MM, Tilp M, Nakamura M, Konrad A. Is muscle stiffness a determinant for range of motion in the leg muscles? Biol Sport. 2024;41(2):115–21. 10.5114/biolsport.2024.131821. 38524826 10.5114/biolsport.2024.131821 PMC10955752

[103] Bojsen-Møller J, Brogaard K, Have MJ, Stryger HP, Kjaer M, Aagaard P, et al. Passive knee joint range of motion is unrelated to the mechanical properties of the patellar tendon. Scand J Med Sci Sports. 2007;17(4):415–21. 10.1111/j.1600-0838.2006.00591.x. 17076834 10.1111/j.1600-0838.2006.00591.x

[104] Koo TK, Hug F. Factors that influence muscle shear modulus during passive stretch. J Biomech. 2015;48(12):3539–42. 10.1016/j.jbiomech.2015.05.038. 26113291 10.1016/j.jbiomech.2015.05.038

[105] Koo TK, Guo JY, Cohen JH, Parker KJ. Quantifying the passive stretching response of human tibialis anterior muscle using shear wave elastography. Clin Biomech (Bristol, Avon). 2014;29(1):33–9. 10.1016/j.clinbiomech.2013.11.009. 10.1016/j.clinbiomech.2013.11.009 24295566

[106] Maïsetti O, Hug F, Bouillard K, Nordez A. Characterization of passive elastic properties of the human medial gastrocnemius muscle belly using supersonic shear imaging. J Biomech. 2012;45(6):978–84. 10.1016/j.jbiomech.2012.01.009. 22326058 10.1016/j.jbiomech.2012.01.009

[107] Deng M, Zhou L, Chen Z, Yuan G, Zhou Y, Xiao Y. An ex-vivo validation of the modulus-length framework to characterize passive elastic properties of skeletal muscle. Ultrasonics. 2023;129:106904. 10.1016/j.ultras.2022.106904. 36463727 10.1016/j.ultras.2022.106904

[108] Berrigan WA, Wickstrom J, Farrell M, Alter K. Hip position influences shear wave elastography measurements of the hamstring muscles in healthy subjects. J Biomech. 2020;109:109930. 10.1016/j.jbiomech.2020.109930. 32807303 10.1016/j.jbiomech.2020.109930 PMC7486790

[109] Kellis E, Blazevich AJ. Hamstrings force-length relationships and their implications for angle-specific joint torques: a narrative review. BMC Sports Sci Med Rehabil. 2022;14(1):166. 10.1186/s13102-022-00555-6. 36064431 10.1186/s13102-022-00555-6 PMC9446565

[110] Hirata K, Yamadera R, Akagi R. Associations between range of motion and tissue stiffness in young and older people. Med Sci Sports Exerc. 2020;52(10):2179–88. 32348099 10.1249/MSS.0000000000002360 PMC7497479

